# Using team-based precision medicine to advance understanding of rare genetic brain disorders

**DOI:** 10.1186/s11689-024-09518-z

**Published:** 2024-03-15

**Authors:** Steven U. Walkley, Sophie Molholm, Bryen Jordan, Robert W. Marion, Melissa Wasserstein

**Affiliations:** 1 Rose F. Kennedy Intellectual and Developmental Disabilities Research Center, Albert Einstein College of Medicine, Bronx, NY 10461 USA; 2 Department of Neuroscience, Albert Einstein College of Medicine, Albert Einstein College of Medicine, Bronx, NY 10461 USA; 3 Department of Pediatrics, Albert Einstein College of Medicine, Albert Einstein College of Medicine, Bronx, NY 10461 USA

**Keywords:** Intellectual and developmental disabilities, Rare disease, Neurodevelopmental disorders, Precision medicine, NextGen sequencing, Disease pathogenesis, Brain

## Abstract

We describe a multidisciplinary teamwork approach known as “Operation IDD Gene Team” developed by the Rose F. Kennedy Intellectual and Developmental Disabilities Research Center (RFK IDDRC) at the Albert Einstein College of Medicine. This initiative brings families affected by rare genetic diseases that cause intellectual and developmental disability together with physicians, basic scientists, and their trainees. At team meetings, family members share their child’s medical and personal history, physicians describe the broader clinical consequences of the condition, and scientists provide accessible tutorials focused on the fundamental biology of relevant genes. When appropriate, possible treatment approaches are also discussed. The outcomes of team meetings have been overwhelmingly positive, with families not only expressing deep gratitude, but also becoming empowered to establish foundations dedicated to their child’s specific condition. Physicians, and in particular the scientists and their trainees, have gained a deeper understanding of challenges faced by affected families, broadening their perspective on how their research can extend beyond the laboratory. Remarkably, research by the scientists following the Gene Team meetings have often included focus on the actual gene variants exhibited by the participating children. As these investigations progress and newly created foundations expand their efforts, national as well as international collaborations are forged. These developments emphasize the importance of rare diseases as windows into previously unexplored molecular and cellular processes, which can offer fresh insights into both normal function as well as more common diseases. Elucidating the mechanisms of and treatments for rare and ultra-rare diseases thus has benefits for all involved—families, physicians, and scientists and their trainees, as well as the broader medical community. While the RFK IDDRC’s Operation IDD Gene Team program has focused on intellectual disabilities affecting children, we believe it has the potential to be applied to rare genetic diseases impacting individuals of any age and encompassing a wide variety of developmental disorders affecting multiple organ systems.

## Introduction

The National Institutes of Health have estimated that there are as many as 10,000 rare diseases, defined as those affecting fewer than 200,000 Americans, yet only a few of these are understood in any detail and even fewer (< 5%) have a known treatment. Many rare diseases affect only a handful of individuals and in some cases as few as one, yet collectively they represent a major unmet medical need for as many as 30 million Americans. One area of major advancement has been in diagnosis. Dramatic improvements in the precision and speed of gene sequencing technology, followed by major reductions in the costs of testing, have radically changed how rare diseases are studied. Whole exome (WES) and whole genome sequencing (WGS), next-generation testing that was so expensive just a decade ago, is now the go-to test in many cases. And while sequencing can provide confirmation of a suspected and well-understood condition, it can also lead to the discovery of a novel and uncharacterized rare or ultra-rare disorder. In these latter cases, parents often go home with a diagnosis consisting of the gene name only, such as *CACNA1A* or *SMARCA2*, and little else given the little-to-no information available to the diagnosing physician.

The speed and ease with which modern gene sequencing can generate a diagnosis stands in sharp contrast to the prolonged effort required to understand how such rare genetic variants cause disease. Indeed, even confirming the pathogenicity of individual variants in a particular gene can be challenging. Overcoming these challenges requires approaches both at the patient level and in laboratory research. For example, unraveling the natural history of a rare condition typically requires years of focused attention by clinicians and parents, yet is invaluable as part of a full understanding of the disease. Likewise, unraveling how rare gene variants induce disease requires years of dedicated effort by scientific researchers from multiple disciplines spanning from cell biologists to cognitive neuroscientists. These efforts often begin with studying how gene variants affect protein expression, structure, and function and are followed by research to determine their impact on specific cell types, metabolic pathways, and ultimately on the function of the brain or other organs. Through such approaches, a fuller picture of the rare disorder begins to emerge, along with insights into the role of the gene in normal cellular function, or even commoner diseases. Possible treatment strategies may also come to light that can be tested in model systems, but again with individual experiments sometimes requiring years to fully execute. It is important to emphasize that at the time of diagnosis, much, if not all, of this essential progress in understanding the disease remains unrealized.

Beyond the significant challenge of receiving limited information from a rare disease diagnosis, parents often will face the dilemma of “what comes next?” even when the child’s immediate medical needs can be met. Many rare and ultra-rare genetic conditions are being diagnosed in children who should be achieving normal developmental milestones, and some of these conditions are progressive. As a result, formulating an understanding of what is known about the condition and its potential consequences for normal development becomes paramount, as is understanding related medical and behavioral issues which might be associated with the condition as the child ages. Equally important is an understanding of whether treatments are available, even experimental ones. To help bridge the gap between a rare genetic disease diagnosis and a comprehensive understanding of the disease, we have instituted a program we call “Operation IDD Gene Team.” This program assembles teams consisting of the parents, their child’s pediatrician, and scientists familiar with the genes and their known biological functions.

### How IDD gene teams are created and how they work

When an ultra-rare, poorly understood neurogenetic condition is identified, typically through WES or WGS, the child’s medical geneticist, who is also a member of the RFK IDDRC, extends an invitation to the family of the affected child. They ask if they would be interested in learning more about their child’s condition by meeting with scientists who specialize in research on the specific gene or related genes. If they agree, we then reach out to our 100-plus RFK IDDRC membership at Einstein/Montefiore for scientists with the relevant expertise and ask if they would be interested in being part of an IDD Gene Team. After identifying the scientific team, the next step is to set up the tutorial which is attended by the family, the scientist(s) and their trainees, the medical geneticist, and any interested clinical fellows or genetic counselors. Although team meetings are most often held in person, they were virtual during the recent COVID-19 pandemic and occasionally when the parents were unable to travel to Einstein.

The IDD Gene Team meeting begins with introductions, followed by the family relaying their child’s story in depth, including their experience with the inevitable “diagnostic odyssey”. The medical geneticist then broadly summarizes what is known about the condition clinically, followed by the researcher presenting a lay tutorial about the science behind the gene and protein/pathway potentially responsible for the child’s condition. If appropriate, current, and future treatment possibilities are discussed. Trainees, including clinical fellows and genetic counselors, as well as medical and graduate students and postdoctoral fellows from the involved laboratory and clinic are encouraged to participate. These meetings typically generate a great deal of back-and-forth discussion and as a result can last 2 h or more and relationships are established that are often sustained. Not infrequently, the participating scientists have gone on to obtain funding through extramural sources, or by a competitive pilot grant program offered by the RFK IDDRC. In several cases, research teams have generated specific model systems that involve the child’s genetic variant. Second Gene Team meetings have also occurred following research advances in the involved laboratory or after the identification of other children with the same genetic disease. Parents have also joined the scientists later in teaching seminars as part of the RFK IDDRC’s training program in IDD or presented in the International Rare Disease Day program that it sponsors annually.

### Examples of IDD gene teams at work

#### SLC17A5 (Salla disease)

The initial IDD Gene Team meeting for *SLC17A5* took place in late 2017 in person at the Rose F. Kennedy Center. The IDD Gene Team consisted of a 3-year-old boy exhibiting neurodevelopmental delay, his parents, the child’s Montefiore medical geneticist, two scientists from Einstein’s Department of Neuroscience whose laboratories are focused on lysosomal disease research, and a member of the RFK IDDRC leadership.

Following introductions, the parents described their family’s experience, noting that their first son was typical in his development but that their second son exhibited developmental delay at 6 months of age, prompting an original diagnosis of cerebral palsy. Later, through WES, it was found that he was heterozygous for bi-allelic pathogenic variants in *SLC17A5*, consistent with Salla disease (SD), a rare lysosomal disorder with no known curative treatment. The mother described in detail how after the diagnosis the child had started multiple types of weekly therapy sessions involving physical and educational activities as well as music therapy. The parents expressed an interest in learning all they could about the disease and how best to help their son.

The medical geneticist next described how variants in *SLC17A5* cause two closely related conditions differing only in age of onset and severity. One was SD, which is slowly progressive and most prevalent in Finland. The other was infantile sialic acid storage (ISSD), a more severe neurodegenerative disorder affecting patients from wider backgrounds. Thus, the disease revealed by WES was rare but certainly not new. The medical geneticist went on to explain that SD had been first noted in 1979 in three children in Salla, Finland (hence the disease name). Each had shown the first signs of developmental delay between ages 6 and 24 months, when they exhibited delayed walking, speech, and motor development. All patients were united by coarse facial features, clumsiness, IDD, ataxia, dysarthria, diffuse EEG abnormalities, and thickened calvaria [1]. An unidentified lysosomal storage disorder was suspected in these original cases because of the presence of vacuolized lymphocytes in peripheral blood smears. Biochemical analysis revealed increased urinary excretion of sialic acid, yet lysosomal hydrolase activity was reported as normal. Progressive cerebellar atrophy and dysmyelination were subsequently observed by MRI [2].

The participating scientists next described that the initial idea of SD being a lysosomal disorder was correct despite lysosomal enzyme activities being normal. This was because the defective protein, sialin, is a membrane-bound lysosomal sialic acid/H + transporter involved in sialic acid efflux out of the lysosome, rather than an enzyme per se [3]. The gene encoding sialin, *SLC17A5*, was cloned in 1999 [4] and around 95% of Finnish SD-patients carry a p.R39C substitution in the gene that affects a highly conserved amino acid just before the first transmembrane domain. This founder mutation has also been found in Swedish and non-Scandinavian patients. No patients with the more severe disease, ISSD, carry this variant. The parents reported that they had no known Finnish or Swedish ancestry, raising further questions about the uniqueness of their situation. Indeed, during this initial team meeting so little was known that there was the consideration that their son might be the only child in the U.S. currently affected by SD.

The scientists went on to explain that while SD is accurately classified as one of the nearly 60 different types of lysosomal disorders, it is not in any of the major lysosomal disease classification groupings (e.g., sphingolipidoses, mucopolysaccharidoses, neuronal ceroid lipofuscinoses), and indeed tended to be listed under “other” in classification schemes, or simply in a group by itself. Most lysosomal disorders are represented by patient support groups or foundations, e.g., the National Tay-Sachs and Allied Diseases Foundation (NTSAD), the National Mucopolysaccharidosis (MPS) Foundation, and the National Niemann-Pick Diseases Foundation (NNPDF). As the scientists reviewed what was known about the molecular basis of SD, it became clear that little recent literature on the gene, protein, or disease existed, other than a complete knockout mouse model made some years previously. SD thus was not only ultra-rare but also truly orphaned as a genetic disorder. The scientists also conveyed to the parents that unlike many other lysosomal diseases that had undergone more extensive research and had established or emerging treatments, there was no known treatment for SD.

As the tutorial was ending a comment was made to the parents that the paucity of information and research on SD might be enhanced if a patient advocacy group or foundation existed. The child’s mother, who had interrupted a promising career to take care of her son, replied that the family simply did not have the time nor the resources to take on such a project, and that their son’s immediate needs were their family’s main concern. However, just 2 months later, she contacted the IDD Gene Team leadership with news that the parents had decided to take on the task of creating a foundation. Shortly thereafter a 501(c)3 was put in place by the family for the Salla Treatment And Research (STAR) Foundation and a website launched (https://www.sallaresearch.org/).

After the creation of the STAR Foundation, the IDD Gene Team leadership helped organize the first combined scientific “Think Tank” and family meeting in 2018. Fifteen scientists and clinicians from 7 research centers who were thought best positioned to make advances in SD research were invited. The STAR Foundation leadership also invited a dozen families from the US and Europe who were discovered after the foundation’s website was created. Fast forwarding to 2023, the “Think Tank” group has grown to 48 scientists and clinicians from 18 centers scattered across the U.S., France, Germany Finland, and the U.K. The group also now has a formalized organization called the Free Sialic Acid Storage Disease (FSASD) Consortium and has clarified the nomenclature surrounding SD and ISSD designations [5]. A second in-person combined scientist-family meeting occurred in concert with STAR Foundation families in October 2023. Today, the STAR foundation and its supporters fund research in 5 of the consortium laboratories, with new cell and mouse models being developed and studied, with an eye on advances in pathogenesis but an emphasis on therapy. Other research support has been generated by FSASD Consortium members from the National Institutes of Health (NIH) through currently funded proposals as well as new grants focused specifically on Salla disease.

#### CACNA1A

The initial IDD Gene Team meeting for *CACNA1A* took place in person in the Rose F. Kennedy Center in late 2018 and a second virtual session was held 2 years later after a second family was identified. The first meeting was attended by the 17-year-old female proband, her parents, two physicians (a Montefiore medical geneticist and a neurologist specializing in seizures), two scientists (a calcium channel expert and an iPSC/organoid specialist), and two trainees (a postdoctoral fellow and a medical student). The second tutorial in 2020 was held virtually and consisted of the above group plus the mother of a second child who through WGS had recently been diagnosed with a *CACNA1A*-related disorder.

Following a welcome and introductions by the RFK IDDRC leadership in attendance, the proband’s mother described her daughter’s history. She noted that early in life, her daughter had shown global developmental disabilities, seizures, and muscle weakness. Later she developed migraine headaches, an ongoing seizure disorder, and problems with balance and walking. Years of testing, including karyotype analysis, nerve conduction studies, electromyography, and a muscle biopsy were not helpful in identifying a definitive diagnosis. By the time her daughter was 10 years old the mother said she had given up hope of finding a cause for her daughter’s condition. But then in 2017, WES was performed and revealed a de novo pathogenic variant in *CACNA1A*. This diagnosis brought great relief to the mother as she thought it would lead to a change in medication and an easier way forward. Yet her geneticist at the time replied that there was little known about this gene and the disease, and that she would be best served by finding a relevant Facebook group. This she did and as a result found other mothers sharing in her dilemma.

After the parents told the story of their daughter and the long diagnostic odyssey, the clinicians reviewed the complex symptomatology that has been reported to accompany pathogenic variants in *CACNA1A*. Termed *CACNA1A*-related disorders, (CRD), these include three different autosomal dominantly inherited neurological conditions: Familial Hemiplegic Migraine, type 1 (FHM1), Episodic Ataxia, type 2 (EA-2), and Spino-Cerebellar Ataxia, type 6 (SCA6)[5, 6]. In addition to these autosomal dominantly inherited conditions, the phenotypic landscape of *CACNA1A* variants includes associations with both epilepsy and cognitive impairments. *CACNA1A* loss-of-function and missense variants have also been demonstrated to cause some epileptic encephalopathies, a group of severe childhood epilepsy disorders, including infantile spasms and Lennox-Gastaut Syndrome [7–10]. In addition to seizures, patients with *CACNA1A* mutations and epileptic encephalopathies showed a variety of cognitive manifestations including psychomotor delay, mild to profound intellectual disability, attention deficit-hyperactive disorder (ADHD), and/or autism spectrum disorders (ASD) [7–9].

The science tutorial following the clinical summary was presented by the calcium channel scientist along with his postdoctoral fellow. Here the team learned that *CACNA1A* encodes the α1A subunit of the neuronal P/Q-type calcium channel. This channel, termed CaV2.1 [11], is critical for synaptic function. Because of this fundamental role in how neurons communicate, it is thought that compromised function of the channel, depending on the variant found in the individual patient, can lead to the remarkable variety of complex clinical states described earlier. Within the brain, these voltage-gated calcium channels (VGCC) are most often found on the presynaptic side of synapses where they regulate neurotransmitter release [12]. Further complicating the pathogenic consequences of this disorder, VGCC containing the α1A subunit have also been reported to be present in lysosomal membranes of cells where they function to facilitate (via calcium release) the fusion of lysosomes with endosomes and autophagosomes [13]. *CACNA1A*-associated channels are strongly expressed in Purkinje cells and cerebellar granule cells. The mouse ortholog of the α1A subunit of the P/Q-type Ca +  + channel is mutated in tottering and leaner mice, two models of absence seizures [14]. In addition to synaptic abnormalities, these mice have also been shown to exhibit defects in lysosomal function and neuronal homeostasis [13].

As the IDD Gene Team meeting progressed and scientists were exposed to the impact of their research beyond the lab through interactions with the patient and her parents, the scientists announced their willingness to make a knock-in mouse model carrying the variant expressed by the proband stating how such a model would be a necessary first step for testing therapies. The parents enthusiastically welcomed the idea.

This first IDD Gene Team meeting on CRDs was followed by several significant developments. Another family was identified by our affiliated medical geneticist whose child had a pathogenic variant in *CACNA1A*, prompting a second IDD Gene Team tutorial 2 years after the first. Here, updates on successful development of the *cacna1a* variant knock-in mouse model were reported. In addition to the two families, a second laboratory joined the meeting, this one specializing in *C. elegans* modeling. They too had generated a model using the first patient’s variant and studies using this model are underway. In addition to these developments, there was also a remarkable update from the first set of parents. The mother, working with another mother in the *CACNA1A*-Related Disorders Facebook group, created the first foundation for this disorder, the CACNA1A Foundation (https://www.cacna1a.org/). This foundation, like the STAR Foundation mentioned above, has expanded rapidly, and developed affiliations with additional scientists, launched fund-raising efforts, and sponsored annual scientific/family meetings.

#### DYNC1H1

The IDD Gene Team meeting for *DYNC1H1* was held in person in the Rose F. Kennedy Center in 2018 and was attended by the 11-year-old proband boy, his parents, the IDDRC leadership, the child’s medical geneticist, two scientists (one focused on dynein and kinesin motor function and the other on in vivo brain disease modeling), and a medical student with interests in this condition. Following introductions, the parents spoke of their son’s global developmental delay that was first noted at about 1 year of age. The parents also spoke of their long diagnostic odyssey that ended with WES in 2018, which revealed that their son had a mono-allelic de novo pathogenic variant in the *DYNC1H1* gene. The parents’ concerns at the time of the meeting focused on their son’s motor planning abilities, coordination, learning difficulties, and hyperactivity. They described an intensive special education program with multiple hours of therapies each week that they developed for him and that they believed had been very helpful.

Following the parents’ presentation, the medical geneticist walked the group through the highly complex disease outcomes caused by different heterozygous mutations in the *DYNC1H1* gene. These include Charcot-Marie-Tooth disease type 2 (CMT2), spinal muscular atrophy with predominant involvement of the lower extremities (SMA-LED), malformations of cortical development (MCD), and severe intellectual disability. The geneticist then described an example of missense variant in *DYNC1H1* that had been shown to be associated with the presence of CMT2 in a family with 23 affected individuals [15]. Members of this family had clinical features including delayed motor milestones, abnormal gait, reduced sensations, and early-onset slowly progressive distal lower limb weakness and wasting. Upper limb involvement was rare and individuals usually remained ambulatory into adulthood. Severely affected family members also noted neuropathic lower limb pains. *DYNC1H1* missense variants have also been identified in several families with SMA-LED, a rare form of autosomal dominantly inherited SMA that principally targets the legs and presents with weakness in early childhood [16]. In addition to these familial syndromes, WES has identified de novo variants in *DYNC1H1* in individuals with intellectual disability [17–19]. Specifically, variants in *DYNC1H1* appear to be an important cause of MCD, a family of neuronal migration disorders that includes lissencephaly, pachygyria, polymicrogyria, and microcephaly. These disorders are associated with severe cases of intellectual disability and involve a disturbance in the coordinated developmental proliferation, migration, or differentiation of specific neuronal populations. Posterior pachygyria is the cortical malformation most associated with *DYNC1H1* mutations, but patients can have additional abnormalities or both pachygyria and polymicrogyria [20].

After the clinical overview, the scientist specializing in molecular motors explained how *DYNC1H1* encodes for a large component of the cytoplasmic dynein complex and that dyneins are a family of cytoskeletal motor proteins that convert the chemical energy of ATP hydrolysis into mechanical work to move organelles and other cargos along microtubules within cells [21]. In the transport system of neurons, members of the kinesin family of motor proteins move cargoes from the cell body into axons and dendrites, while cytoplasmic dynein transports them back. In the brain, these motor proteins play critical roles in numerous cellular processes, particularly retrograde axonal transport, nuclear positioning, Golgi localization, and autophagy. To elucidate how mutations in the dynein molecule result in diverse clinical features, the scientist presented a video illustrating the dynein molecule transporting cellular organelles along microtubules. Some heterozygous pathogenic variants, like the one identified in the proband, lead to only slight deviations from typical movement, while others can cause significant functional disruptions. This pivotal role of dynein in various cellular functions explains the range of neuropathies and brain malformations seen in individuals with pathogenic variants in DYNC1H1.

As with other IDD Gene Team meetings, this first tutorial was followed by many other interactions with the family. The entire family, including the proband’s siblings, attended the 2019 Rare Disease Day event at Einstein sponsored by the RFK IDDRC. Here, they were interviewed by their son’s pediatrician in front of a large audience where they shared their story. The dynein scientist then reviewed how the dynein motor works in cells, particularly in neurons. In 2022, both scientists involved in the IDD Gene Team were awarded RFK IDDRC pilot grants to pursue studies on how the patient’s variant can alter the function of dynein. The first uses single-molecule fluorescence and optical trapping assays to study dynein function, a study that subsequently received NIH funding. The second study is directed at the development of a knock-in mouse model which again is focused specifically on the proband’s variant. In late 2022, the family re-joined the scientist in a virtual IDD trainee seminar sponsored by the RFK IDDRC.

#### PPM1D (Jansen de Vries Syndrome)

The IDD Gene Team meeting for *PPM1D* occurred in person in the Rose F. Kennedy Center in 2017. The proband and his parents attended the session, along with his Montefiore-based medical geneticist, a scientist with expertise in iPSC/organoid modeling and an interest in this gene and pathway, and members of the RFK IDDRC leadership. After introductions by the IDDRC leadership, the parents talked about their son’s early years leading up to the diagnosis. This was further amplified by the medical geneticist who had initially seen the child at 16 months of age because of developmental delay and hypotonia. What emerged in the discussions was that he was born at full term after an uncomplicated gestation but had had difficulties with feeding from the newborn period, which were attributed to his generalized hypotonia. Feeding was also complicated by gastroesophageal reflux disease. His motor development had been globally delayed: he had rolled over (belly to back) at 6 months, and at 16 months required assistance to stand independently. Though he made sounds, his only words were “mama” and “dada.” In one exam at that time, he was noted to be below 5th percentile for height and weight, with head circumference within the normal range. He had slightly dysmorphic facial features (low set, posteriorly rotated ears, small epicanthal folds, thin upper vermilion border of the lip, and a generalized coarsening of the facial features), small hands and feet, hypotonia, and extreme sensitivity to noises. Testing at the initial evaluation using microarray comparative genomic hybridization revealed a small deletion in chromosome band 17q. Parental studies confirmed that the deletion was inherited from the unaffected father and therefore unlikely to be pathogenic. DNA analysis for fragile X was negative. As the years passed the child’s development continued to be slow and he manifested a series of behavioral abnormalities, including ADHD, hyperacusis, and sensory integration issues. The definitive diagnosis finally came with WES showing a de novo pathogenic mutation in *PPM1D.*

Following the report from the parents, the medical geneticist then spoke about the clinical consequences of pathologic variants in *PPM1D*, noting that this condition had first been identified in 2017 and known as Jansen-de Vries syndrome. Review of the literature revealed one individual with similar phenotypic features who had the same pathogenic variant mutation (subject 4 in Jansen, et al., 2017) and that individuals with variants in this gene can manifest a wide spectrum of phenotypic features. Deep phenotyping of individuals with IDDs linked to truncating PPM1D variants has revealed a syndrome typified by cognitive impairment, gastrointestinal difficulties, and a high pain threshold. In addition to mild to severe intellectual disability, most individuals from this cohort had behavioral problems ranging from anxiety to constellations of behaviors associated with autism spectrum disorder, and half of all individuals showed hypersensitivity to sound. Other common conditions included hypotonia, short stature, feeding difficulties, periods of illness with fever and/or vomiting, vision problems, and small hands and feet with brachydactyly [22].

The scientist next presented a lay summary of the gene and the protein it encodes, and how its absence might be related to developmental and intellectual disabilities. It was explained that *PPM1D* encodes for Mg2 + /Mn2 + -dependent protein phosphatase 1D, an enzyme that contains an N-terminal phosphatase domain and a C-terminal nuclear localization signal that would likely target the protein to the nucleus. When activated, the enzyme dephosphorylates and inhibits p53 and other tumor suppressors. In so doing, the enzyme regulates the DNA damage response and other cellular stress-response pathways [22–24]. Gene-expression databases show that *PPM1D* is expressed in both the fetal and adult brain. It appears to be more widely expressed during development, with transcripts also found in fetal liver and skeletal muscle. The scientist went on to note that the variants in *PPM1D* that are associated with intellectual disability are in the last or penultimate exons of the gene and are predicted to generate a premature stop codon. This results in a truncated protein that retains catalytic activity. However, the half-life is extended because of the loss of a proteasomal targeting and degradation signal at the C-terminal end of the protein [25]. This leads to prolonged downregulation of p53 and other tumor suppressors. A nuclear targeting element is also lost [22].

Toward the end of the tutorial, the scientist explained how iPSC methodology can be used to explore how specific mutations in genes like *PPM1D* can cause disease. The parents responded enthusiastically and agreed to work with the scientist in this effort. They later raised funds through their child’s school and other activities to support *PPM1D* research in the scientist’s laboratory, a story that was publicized by a local TV station. As a result of the family’s support, along with a pilot grant from the RFK IDDRC provided in 2019, the scientist generated iPSCs from the proband and his typically developing brother, and constructed CRISPR-edited iPSC lines containing a null mutation like that found in the proband. He also generated the same variant in a neuroblastoma cell line using CRISPR. These studies subsequently received NIH support.

As with other IDD Gene Teams, the connection between the family and the scientist continued, with the family participating in RFK IDDRC’s 2018 Rare Disease Day program. In 2022, the Jansen de Vries Syndrome Foundation, of which the parents are members, named the IDD Gene Team scientist to their Medial Advisory Board. In 2023, the family also participated in a virtual RFK IDDRC trainee seminar hosted by the scientist.

#### COL4A1 (Gould Syndrome)

The *COL4A1* IDD Gene Team initially met in 2019, with a follow-up meeting at the request of the parents in 2023. Both meetings were held in person in the Rose F. Kennedy Center. The first Gene Team meeting consisted of the proband, who was 4 years old at the time of the meeting, her parents, the child’s medical geneticist, 3 members of a neuroscience laboratory (lead scientist and 2 postdoctoral fellows) specializing in cell-cell interactions and basement membrane function, and a representative from the RFK IDDRC leadership. For the second meeting in 2023 a maternal aunt also joined, along with a Medical Student Training Program (MSTP) student from the neuroscience laboratory.

During the first session, the proband sat quietly cradled in the mother’s lap. Following introductions by the RFK IDDRC leadership, the mother told the story of her daughter. Born at 27 weeks gestation, she had manifested failure to thrive and had slowly lost her ability to see. The medical geneticist volunteered additional details, including that at present she weighed only 14 pounds (< 5th percentile; the typical weight of a 6-month-old), had microcephaly, global developmental delay, bilateral congenital cataracts and glaucoma, seizures, and spasticity. It was noted that she had low vision, was unable to sit independently due to spasticity, and expressed only a few, single words. It was also explained that an MRI performed when she was in her first year showed periventricular leukomalacia, a small right caudate lacunar infarct, a thin corpus callosum, brain asymmetry, and the suggestion of several “prominent venous structures”. In 2018, WES revealed that the proband was heterozygous for a de novo pathogenic variant in *COL4A1.*

The proband was 7 years old at the time of the second IDD Gene Team meeting and although still legally blind, she wore contact lenses for corrective vision. She was still unable to sit independently and had difficulty holding her head up without support. During this session, she again was supported in one of the parents’ laps but played quietly with an iPad throughout the session. The mother explained that she relies on a stroller/wheelchair for mobility and that she can press large buttons with her fist and nose and in this way can navigate her iPad and a 9-panel assisted communication device. She can speak five words, among which include, “hi”, “mom”, and “hello”. The mother further explained that after placement of a gastric feeding tube and improvements in her diet that her daughter gained additional weight, and now weighed 25 pounds (< 5th percentile). She was being regularly followed by a multidisciplinary team covering ophthalmology, neurology, gastroenterology, and pulmonology medicine, and was receiving numerous medications to control glaucoma and seizures, and to improve her gastrointestinal issues. She was attending a school in New York City that focused on educating students with special needs from age 5 through 21. The proband’s mother stated how much her daughter enjoyed attending school and described her daughter’s attitude as being independent.

Further discussion by the medical geneticist indicated that mono-allelic pathogenic variants in *COL4A1* are usually highly penetrant and manifest clinically as a spectrum of disorders, described in the literature as affecting fewer than 100 families. Variants in a second gene, *COL4A2*, can also cause overlapping clinical and pathologic outcomes. Together, the condition caused by variants in these two genes are referred to as Gould Syndrome [26]. Despite being inherited in an autosomal dominant fashion, more than a quarter of patients with *COL4A1*-related diseases harbor a de novo variant and exhibit variable age of onset both within and between families. Clinical outcomes of *COL4A1* variants are highly varied, tend to be multi-systemic, and are often severe. Most frequently they are characterized by the presence of abnormal blood vessels in the brain, developmental defects of the eye, myopathy, and kidney abnormalities. Consequences emanating from the brain microvascular abnormalities are highly variable and can range from brain hemorrhage before and after birth leading to periventricular leukomalacia, porencephaly, lacunar infarcts, and other changes. Clinical findings can include infantile hemiparesis, seizures, single/recurrent hemorrhagic stroke, ischemic stroke, isolated migraine with aura, and significant intellectual/ developmental delay.

During the two IDD Gene Team meetings, a postdoctoral fellow and MSTP student outlined the known science behind the gene. The Gene Team participants learned that *COL4A1* encodes Col4, an alpha-1 chain of type IV collagen, which is an essential non-fibrillar or mesh-like protein of the extracellular matrix (ECM) and is critical for the formation of basement membranes (BMs) of cells [27]. It is well documented that the formation of BMs is a prerequisite for normal development and function of most types of cells in many organs [28]. Individual components of BMs are known to regulate different biological activities, such as the development, proliferation, differentiation, growth, and migration of cells, which requires cell surface receptors. As such, BMs control cellular functions by binding to and modulating local concentrations of growth factors and cytokines. BMs have also been shown to regulate cell polarity and cell migration. Given this wide variety of functions, it is not surprising that the consequences of a defect in this key BM constituent (type IV collagen) could lead to a wide spectrum of clinical consequences.

During the close of the second Gene Team meeting, team leadership shared that there was a nonprofit organization (the Gould Syndrome Foundation) designed to help families affected by variants in *COL4A1* (as well as the closely related *COL4A2* gene) through education, advocacy, and a global patient registry to bring research and medical therapeutic options to those affected (https://gouldsyndromefoundation.org). The lead scientist then revisited his suggestion made at the first COL4A1 team meeting, that generating a knock-in mouse model using the variant discovered in the family’s daughter would be important to better understand her condition. He reported that, with the help of a pilot grant he received after that first session from the RFK IDDRC, that his lab had successfully accomplished this and that this new mouse model was currently being characterized and additional studies planned.

#### KDM5C (Claes-Jensen type, X-linked Intellectual Disability)

Although most of our IDD Gene Team meetings have been triggered by clinicians seeking help with a family, occasionally, a meeting will be requested by another member of the IDDRC program. In late 2019, an MSTP student working on the function of the *KDM5C* gene in fly models, approached the RFK IDDRC leadership asking whether a family impacted by variants in this gene might be invited to the Rare Disease Day event planned for late February 2020. The student had become interested in the phenotype of children who were hemi- or heterozygous for pathogenic variants in this X-linked gene. He had joined a *KDM5C* Support Group’s Facebook page and having attended the RFK IDDRC-sponsored Rare Disease Day event the previous year, knew it worked much like the IDD Gene Team program. That is, a family is brought together with a pediatrician and a scientist to discuss the genetic condition affecting their child, with the only difference being that an audience of 100 + is looking on. The IDDRC leadership embraced the idea, and the family was invited to participate. Much to our surprise and delight, after the KDM5C Facebook group heard of the invitation, eleven additional families from across the U.S. and one from the U.K., reached out and asked to attend the event.

On the morning of Rare Disease Day in February 2020, twelve *KDM5C*-impacted families, including parents, affected children, and their unaffected siblings, arrived at Einstein and met face-to-face for the first time. To accommodate their needs, the RFK IDDRC leadership solicited help from Einstein’s Rose F. Kennedy Children’s Evaluation and Rehabilitation Center (RFK CERC) to create a morning program presented by multiple professionals specializing in developmental pediatrics, occupational and physical therapy, genetics education, pediatric endocrinology, and neurology. The resulting session allowed the families to share their stories and to ask any questions they had about *KDM5C* variants affecting their children. During this morning session, the RFK CERC leadership also provided the families with results of a questionnaire that had been sent to families in the KDM5C Facebook group several weeks before the Rare Disease Day meeting. Caregiver-reported data for 37 unrelated individuals with pathogenic variants in *KDM5C* were summarized, which represented the largest cohort of such children reported to date. Analyses confirmed earlier findings for males with *KDM5C* variants showing moderate to severe ID with significant syndromic comorbidities such as epilepsy, short stature, and craniofacial abnormalities. Importantly, the questionnaire also revealed the importance of this X-linked condition in heterozygous females, with 70% displaying syndromic features including gastrointestinal dysfunction and hearing impairment, and more than half with a diagnosis of autism spectrum disorder (ASD) or features consistent with this condition. Overall, this analysis provided further evidence of sexually dimorphic heterogeneity in disease presentation and suggested that pathogenic variants in the *KDM5C* gene might be more common than previously believed [29].

In addition to the panel of professionals who presented during the morning session, the mother mentioned earlier in the summary of the *SLC17A5* IDD Gene Team who had established the STAR Foundation for Salla disease was invited to this session to provide the *KDM5C* parent group with insights and advice on how to start of their own foundation.

Following this morning session, the regular Rare Disease Day program was held, which consisted of several families with children with a rare disease, including one of the families that had participated in the morning session. Joining this family in a presentation were the MSTP student who had initiated the original invitation and his laboratory mentor who was directing the research on *KDM5C*. As the family was interviewed by the student, the audience learned of their long diagnostic odyssey and ultimately the value of WES in finding a diagnosis for their daughter. It was explained that being X-linked, variants in *KDM5C* primarily are known to cause disease in hemizygous males and that the phenotype in heterozygous females is more variable and milder and thus easily overlooked. The morning activities were also summarized for the larger audience. This included discussion of findings from the *KDM5C* Facebook questionnaire showing that autism-like features occurred in some females, suggesting that *KDM5C* variants being responsible for ASD features in females may be underestimated.

After the parents’ session with the MSTP student, the scientist gave an overview of her work and that of others on this gene and what it was believed to do in cells [30]. It was reported that the gene encodes an enzyme known as lysine demethylase 5C, which is a protein that plays a vital role as a transcriptional regulator [31]. The KDM5C protein is known to be ubiquitously expressed, with its highest levels found in brain in humans [32]. Within the mouse brain, it is expressed within neurons and astroglia in regions including the hippocampus, cortex, amygdala, and cerebellum [33]. In non-neuronal cells, it has been shown to function as a transcriptional co-repressor of the RE1-silencing transcription factor (REST) complex to prevent the misexpression of neuronal genes [34]. Importantly, a Kdm5c knockout mouse model has been generated and found to display various behavioral deficits parallel to those in patients, such as learning and memory impairments and decreased seizure thresholds. One focus of interest with this model has been to determine the impact of KDM5C variants on dendritic spines, alterations in which are often a feature observed in many forms of intellectual disability. Studies in rat cerebellar granular neurons and mice pyramidal neurons of the basolateral amygdala demonstrate that *Kdm5C* knockout results in abnormal dendritic spines [35]. Collectively, although these studies provide strong evidence that disruption of *KDM5C* may impact programs critical to neuronal development including dendritic spine formation, either in a demethylase dependent or independent manner, the precise mechanism(s) relating KDM5C dysfunction to intellectual disability remain largely unknown.

As with other IDD Gene Team meetings, interactions between the families and Einstein physicians and scientists have continued. Early in 2023, the family interviewed during the Rare Disease Day session returned, this time virtually, to join the scientist in an RFK IDDRC-sponsored T32 teaching seminar on KDM5C.

### The value of parent empowerment

Rare diseases, particularly those impacting children and compromising their intellectual, behavioral, and physical development, place a tremendous burden on their families. Family resources to help the child can be stretched thin, and gaining assistance from social services, schools, and the educational system is often challenging. The medical establishment itself has its own limitations. While modern diagnostic tests like WES and WGS have revolutionized the ability to determine the underlying cause of an individual’s rare genetic disease, the answer all too often is a series of letters and numbers constituting a gene name whose function may be little understood, if at all. Parents going home with a diagnosis of their child having a KDM5C variant, or Claes-Jensen type, X-linked Intellectual Disability, may feel lost, particularly if their physician lacks expertise about the condition. And given the rarity of most of these newly identified genetic diseases, it is only understandable that the frontline healthcare provider might be at a loss to do more.

A primary goal of the IDD Gene Team program has been to overcome this dilemma by empowering parents. This is accomplished by providing an array of information about their child’s condition that includes scientific and clinical knowledge, and discussion of how these come together to shape anticipatory guidance and inform therapeutic options. Importantly, this information is presented in an accessible and understandable manner, ensuring that parents can make informed decisions even when there is no standard prognosis and treatment protocol in place. Critically, a true dialog among researchers, clinicians, and families occurs in these meetings and each party’s perspective is enriched. Beyond providing information, a key aspect of this empowerment is letting parents know that they are not alone in their journey. It emphasizes that physicians genuinely care about their child’s well-being and that there are dedicated scientists actively working on understanding the specific condition affecting their child. The outcomes of our IDD Gene Team efforts to empower parents have been uniformly positive. To date, no less than three foundations have been created through the efforts of parents—notably mothers—who were part of this program, these being for *SLC17A5*, *CACNA1A*, and *KDM5C*. This is not to say that the driver for each of these individuals was solely their interaction with our program, but given their own testimonials (https://www.einsteinmed.edu/docs/centers/iddrc/iddrc-newsletter-winter-2023.pdf), it is clear that the experience they had as part of “the team” was a major contributor.

As mentioned earlier, the parents of the proband with Salla disease (*SLC17A5*) created the STAR Foundation (https://www.sallaresearch.org) in 2018 which today has its own scientific advisory board that includes two of the Einstein faculty who were part of their IDD Gene Team. This foundation supports a major research effort on Salla disease at the NIH, along with smaller projects in laboratories in the U.S. and France. This is a remarkable transformation considering that in 2017, there was little evidence of clinical or laboratory research focused on this disease. Current research includes the development of new mouse and cell models, an ongoing natural history study, and a patient registry in development. Research is also exploring the function of sialin, a key component in understanding and treating the disease. Notably, there is significant research on therapies aimed at restoring the loss of sialin function, including pharmacological as well as gene-based approaches. The foundation also organizes periodic Sunday morning Zoom conferences during which families from the U.S., Canada, the Dominican Republic, Finland, France, the U.K., Switzerland, and Germany come together to share their experiences. It provides a homebase for the FSASD Research Consortium scientists and physicians who sprang from the first international “think tank” on Salla disease organized by IDD Gene Team members in 2018. The foundation also organizes and sponsors joint scientist-physician-family conferences, the second of which occurred in the latter part of 2023. What began in a modest conference room in the Rose F. Kennedy Center in 2017 has grown to an international effort to fully understand and treat Salla disease. It is a testament to the determination of one parent that was empowered with the idea that it could be done.

The story of *CACNA1A* has followed a similar course. Three years after her 20-year-old daughter was finally diagnosed with a *CACNA1A* variant and 2 years after participating in an IDD Gene Team and joining a CACNA1A Facebook group, this mother launched the *CACNA1A* Foundation (https://www.cacna1a.org/). With remarkable speed, this foundation has become fully established with its own Scientific and Medical Advisory Board and an ambitious program for supporting basic research in search of treatments, a patient registry, biobanking, awareness raising, and public outreach. It has also networked successfully with other rare disease organizations and has supported annual family-scientific conferences since 2021. In a significant recognition of its dedication and potential impact, the foundation was awarded a prestigious Rare as One grant from the Chan-Zuckerberg Initiative in 2021. Like the STAR Foundation, this story revolves around the determination and vision of a single mother who was inspired and empowered to take action. It serves as a testament to the remarkable impact that one individual’s dedication can have in the world of rare diseases.

Perhaps most dramatically, the *KDM5C* story brought together a dozen families with children affected by variants in this gene who met in person for the first time in 2020 at a Rare Disease Day conference at Einstein. On that day, those families experienced a Pay It Forward moment, when the mother who had established the STAR Foundation shared with them the step-by-step process she had taken to create a foundation. As a result, within 2 years, the *KDM5C* Advocacy, Research, Education and Support (KARES) Foundation (https://kares.foundation) was launched. Like the other two foundations mentioned above, KARES is focused on family engagement, raising funds to both research and promote public awareness of this rare disease. Once again this shows the importance of one parent’s empowerment infused into the greater KDM5C community.

### The outcome for physicians, scientists, and their trainees

The impact of the IDD Gene Team experience has not been limited to the participating families. The physicians and scientists who work with the families have also been transformed by what they experience. Like the parents, the physicians and participating clinical fellows learn more about the science behind the disease and in some cases may be inspired to seek out other families in their clinic with similar conditions. Exploring the complexities of one rare disease also equips medical professionals to better support families with unrelated rare conditions, broadening their expertise. Scientists and their trainees have also been significantly impacted by the Gene Team experience. Unlike medical professionals who are regularly exposed to the struggles and hardships experienced by individuals and families affected by rare diseases, scientists often operate in isolation from such real-life experiences. However, after participating in the IDD Gene Team sessions, their perspective on the genes or proteins they study in their laboratories undergoes a profound transformation. These molecules can no longer be viewed in the same detached manner as before as the discoveries made about the gene or protein are now connected to cells in the child they met during the IDD Gene Team session. This transformation turns what in one moment might be considered esoteric knowledge into direct, pragmatic insights into a disease and a human life. As the laboratory expands its research, this may also trigger possible avenues toward therapy that had not been considered previously. Trainees are also deeply affected by the experience of being part of an IDD Gene Team. They may be so moved by their experience meeting affected children and their parents that they dedicate their careers to this or other rare diseases. In addition, the scientists and physicians involved in the IDD Gene Teams have often been invited to be members of the scientific and medical advisory boards of foundations, thus bringing them into the disease community in a larger way.

An understanding of the involved rare disease can also be significantly impacted. Natural history studies and patient registries growing out of the efforts of newly organized foundations can be transformative for understanding the disease. Of critical importance, an understanding of the disease progression in many affected children can prepare the ground for later clinical trials. Like the physicians, IDD Gene Team scientists and their trainees have been inspired to go further in their research on the child’s genetic variant and the encoded protein. Often, they have created new cell, worm, fly, or mouse models using the patients’ actual variants, to determine their disease impact more precisely and as a means for testing therapeutic approaches. As part of the RFK IDDRC’s Gene Team program, pilot grants are competitively awarded to research laboratories that can aid in jumpstarting new research directions. These are often followed a year or two later by the awarding of major grants by the NIH. As the research expands and publications appear, other interested scientists and physicians worldwide can become recruited into the effort. No longer are the disease and the impacted families left orphaned and alone.

It is worth highlighting that understanding how a gene contributes to a disease can be a powerful means of understanding that gene product’s physiological function. This knowledge can entice researchers focused on unraveling the complexities of the brain to tackle rare diseases. Research directed at a rare disease can generate new knowledge about how the human body functions. These insights may in turn provide new ways of thinking about more common diseases, including therapies for those diseases. Thus, whether the focus is on parents and their children, on physicians, scientists, and their trainees, or on advances in scientific knowledge and medical care, the value of enhancing research on rare diseases simply cannot be understated.

### Practicality and scalability of the IDD gene team program

Our experience with generating IDD Gene Teams suggests that such a program could be established in any academic medical center, and indeed, given the IDD research mandate of the national network of IDDRCs, they would be ideal settings for such activities. Then again, the program certainly need not be focused only on IDDs, but rather could be implemented for rare genetic diseases of all types. To date we have identified 23 genes and conditions for which we wanted to develop IDD Gene Teams and for 12 of these we have successfully launched the meetings. Limitations are primarily finding labs at Einstein with appropriate expertise and interest in joining the team rather than families not being interested. For the latter, in only one circumstance did we have a family who was invited to participate withdraw from the process. Educational levels of the families have not appeared to impact the success of the meetings, with those lacking a completed high school education being as equally receptive and grateful as those who are college-educated. It is understandable that families with greater resources would be more likely to take the initiative to develop foundations, which has been the case. Another concern we had with our program early on was whether scientists would be able to pitch their explanation of the involved gene, protein, and disease pathway in a suitable manner. As a result, a member of the IDDRC leadership routinely meets with the scientists and their trainees before the gene team meeting to review their slides. Results have been truly gratifying and without exception the tutorials have been exceptionally well presented by the scientists or their trainees. Most of our team meetings to date have been in person, and while preferred simply because of the enhanced face-to-face interactions, virtual or hybrid meetings when necessary have also proven successful. An overarching aspect of the team meetings we have had to date is how everyone in attendance benefits. Parents are truly grateful and empowered, clinicians gain science colleagues and a greater understanding of their patient’s condition, and scientists and their trainees come to see their research as relevant to the human condition, often expanding their efforts on the exact gene variant impacting the child.

## Conclusions

The availability of Next-Generation Sequencing has dramatically increased the identification of previously unknown disease-causing genetic variants. While an exciting development at the front end of precision medicine for rare and ultra-rare diseases, these newly discovered variants present uncharted territory for the families of children with rare diseases, as well as for their healthcare providers. Understandably, parents often take on the burden of responsibility for their child’s unexplained disease, and yet a definitive diagnosis in the form of a gene name, without a fuller explanation, will prove inadequate for most parents. Healthcare providers too, may be left wondering about the adequacy of the diagnosis they have provided. We have found that the collaborative atmosphere created in a Gene Team that includes basic scientists together with parents and healthcare providers can help overcome both dilemmas. Parents are empowered by what they learn, and clinicians gain science partners in understanding the revealed disease. Importantly, the scientists are also transformed and often in turn transform their research plan to include the discovered gene variant in their studies, fulfilling a critical goal of precision medicine. New discoveries emerging from the study of rare diseases are also transformative, providing insight into more common disorders as well as normal organ function. Finally, the Gene Team meeting creates an inspirational learning experience for trainees, both clinical and scientific, thus fostering the next generation of critical players in the healthcare system. In sum, the Gene Team approach provides one model for how to bring together stakeholders with unique perspectives to facilitate a better outcome to the process enabled by modern genetic testing, ultimately advancing greater understanding and treatment of rare diseases, for the benefit of all.

### Box insert

Advantages of a team-based approach to rare genetic disease.

1. Parents/care givers/individuals are empowered with greater knowledge of the genetic condition impacting their children.
2. Families and individuals are made aware that scientists as well as physicians are invested in helping in understanding and treating such conditions.
3. Families and individuals empowered by their experience on the team often go on to make major contributions to public awareness and understanding of the disease. Included here are the creation of internet-based disease-focus groups as well as foundations.
4. Team physicians expand their understanding of the cause of the condition and are better able to guide both the parents/individuals and the scientists in their efforts.
5. Clinical fellows and trainees can be inspired by the unfolding process and dedicate their efforts to this and similar conditions.
6. Scientists and their trainees are given real-life experiences of the conditions they are researching in their labs.
7. Trainees in research laboratories can have career-shaping experiences as they learn the importance of translational research as well as the value of their efforts beyond the laboratory. What before may have been considered esoteric information about changes in protein folding caused by a particular gene variant comes alive with consequences.
8. After their experience, Gene Team scientists may redirect their lab’s efforts to focus on the specific disease or its causative gene variant, expanding efforts to secure additional research funding and facilitating greater knowledge about a little-known genetic disorder.
9. Knowledge gained from the study of rare diseases can lead to new insights into how the normal body works, or into commoner diseases and their treatment.
10. Research collaborations worldwide fostered by foundation efforts and outreach by the involved scientists, provide for the possibility of major breakthroughs in understanding and treatment of a what were previously little-known and understudied orphan diseases.

## Data Availability

All results are provided in the manuscript.
